# Interspecies Gene Name Extrapolation—A New Approach

**DOI:** 10.1371/journal.pone.0138751

**Published:** 2015-09-25

**Authors:** Roxana Cojocneanu Petric, Cornelia Braicu, Cristian Bassi, Laura Pop, Ionelia Taranu, Nicolae Dragos, Dan Dumitrascu, Massimo Negrini, Ioana Berindan-Neagoe

**Affiliations:** 1 Department of Biology, Babes Bolyai University, Cluj-Napoca, Romania; 2 Research Center for Functional Genomics, Biomedicine and Translational Medicine, Iuliu Hatieganu University of Medicine and Pharmacy, Cluj-Napoca, Romania; 3 Department of Morphology, Surgery and Experimental Medicine, Ferrara University, Ferrara, Italy; 4 National Institute of Research and Development for Biology and Animal Nutrition, Balotesti, Romania; 5 Taxonomy and Ecology Department, Institute of Biological Research, Cluj-Napoca, Romania; 6 2nd Department of Internal Medicine, Iuliu Hatieganu University of Medicine and Pharmacy, Cluj-Napoca, Romania; 7 Department of Morphology, Surgery and Experimental Medicine, Ferrara University, Ferrara, Italy; 8 Laboratory for Technologies of Advanced Therapies (LTTA), Ferrara University, Ferrara, Italy; 9 Department of Experimental Therapeutics, MD Anderson Cancer Center, Houston, Texas, United States of America; 10 Department of Functional Genomics and Experimental Pathology, The Oncology Institute “Prof. Dr. Ion Chiricuta”, Cluj-Napoca, Romania; 11 Department of Immunology, Iuliu Hatieganu University of Medicine and Pharmacy, Cluj-Napoca, Romania; University of North Carolina at Charlotte, UNITED STATES

## Abstract

The use of animal models has facilitated numerous scientific developments, especially when employing “omics” technologies to study the effects of various environmental factors on humans. Our study presents a new bioinformatics pipeline suitable when the generated microarray data from animal models does not contain the necessary human gene name annotation. We conducted single color gene expression microarray on duodenum and spleen tissue obtained from pigs which have been exposed to zearalenone and Escherichia coli contamination, either alone or combined. By performing a combination of file format modifications and data alignments using various online tools as well as a command line environment, we performed the pig to human gene name extrapolation with an average yield of 58.34%, compared to 3.64% when applying more simple methods. In conclusion, while online data analysis portals on their own are of great importance in data management and assessment, our new pipeline provided a more effective approach for a situation which can be frequently encountered by researchers in the “omics” era.

## Introduction

When *in vitro* studies can no longer offer researchers reliable information to increase scientific knowledge beneficial to humans, the use of animal models becomes a natural step in the process [1]. Choosing the right model is crucial, especially when the information that is generated is of great importance to human health, but the experiments cannot be performed on human tissues. Such a case is our study which attempted to find the effects of the co-contamination with two common agents on the digestive tract, and on the organism as a whole, using the microarray gene expression technology. Specifically, we studied the molecular processes that take place in cells that belong to the gastrointestinal (GI) tract when exposed to the effects of Zearalenone–a common mycotoxin produced by mold species from the genus *Fusarium*–either alone, or in combination with a common bacteria, *Escherichia coli*, which is either commensal or, in certain situation, can become pathological [2–4].

As an animal model we chose the pig, because of the histophysiological–and consequently pathology-related resemblance between *Sus scrofa* and *Homo sapiens* [5, 6]. Thus, the results generated by this experiment has the potential to offer information about the effects of this co-contamination in humans by converting the differentially expressed porcine transcripts to their human orthologues.

The limitation that we encountered was due to the fact that the gene panel that we used was not a commercial one but a custom one. The use of custom panels for structural and functional molecular assays has both advantages and disadvantages. The most obvious benefit is the fact that the analyzed transcripts can be chosen in such a way as to best fit the needs of researchers, but the downside may consist in lack of extensive annotations and complete extrapolation possibilities. We were faced with this challenge, since only a fraction of the probe sequences had a corresponding gene name that belonged to a common database.

The process of transcript name extrapolation from pig to human using online data mining tools is tedious and lengthy, particularly when it involves tens of thousands of features, such as in the case of microarray probes. At the same time, the use of public servers also involves limitations concerning the amount of data that can be uploaded for analysis at one time. To exemplify, BioMart is an online suite of tools provided by Ensembl [7, 8] which offers, through a friendly and easy to use interface, the possibility to perform many different queries necessary for genomic analyses, such as rapid and customizable retrieval of data associated with genes. One of the applications can compare items from a list–such as probe IDs–to different databases containing various types of genomic entities (genes, proteins, microRNAs, other transcripts) from most available nomenclatures (eg. COSMIC, Kazusa, VEGA, UNIPROT, etc.). The list of items can be either uploaded as a text file or can be copy-pasted directly into the browser, and the user also has the possibility to select the species for which the genome enquiry is conducted. Still, since this is an online application which can be access simultaneously by many users from across the world, there are traffic limitations which are necessary to insure the proper functioning of the service. The platform recommends that the input do not contain more than 500 elements. While for usual interrogations this might not be a problem, for situations such as identifying the corresponding names for a full set of microarray probes–almost 60,000 different probe IDs in our case–it would require considerable time to complete, since each enquiry needs to be run against many different databases. Moreover, our custom panel contained probe identification names from different nomenclatures, most of which were not present in common databanks.

Therefore it was mandatory to elaborate a functional bioinformatics pipeline in order to correctly identify the human equivalent genes for the transcripts that displayed significant differential expression in the treated samples compared to the untreated controls. Thus, we performed a series of alignments and conversions using different web-based as well as command line freeware tools and programs, which generated the human orthologues necessary for downstream analyses.

## Materials and Methods

The biological samples used to perform the microarray experiment that generated the differentially expressed transcripts for which we accomplished the porcine to human gene name extrapolation consisted in 15 spleen and 15 duodenum tissue samples collected from weaned pigs. The animals had been exposed after weaning to an experimental contamination with zearalenone (100 ppb ZEA) and *Escherichia coli*, either as single contaminating agents or in combination. At the end of the experiment, pigs were stunned and slaughtered in an EU-licensed abattoir according with the EU Council Directive 2010/63/CE. Organ samples were collected on ice from all animals, weighed and stored at –80°C until the analyses. Animals were cared for in accordance with the Romanian Law 206/2004 and Romanian governmental decision 28/2011 for handling and protection of animals used for experimental purposes. The study protocol was approved by the Ethical Committee of the National Research-Development Institute for Animal Nutrition and Biology, Balotesti, Romania.

The distribution of the samples used for the microarray experiment, in relation to treatment, is presented in Table 1. The tissue samples were mechanically disrupted using a Polytron homogenizer, with 800 μl TRI Reagent (Sigma-Aldrich) added to each tube, and extraction of total RNA was performed according to the manufacturer’s phase separation protocol. The RNeasy Micro Kit form Qiagen was used to purify the extracted RNA, which further underwent qualitative and quantitative evaluation using the Bioanalyzer 2100 (Agilent Technologies) and NanoDrop-1000 (Thermoscientific). After dilution and cDNA synthesis, probes were generated and hybridized in concordance with the Agilent recommended protocol for one color gene expression experiments (Agilent manual: G4140-90040), using 600 ng of cRNA-Cy3 probe for each sample.

**Table 1:** Sample distribution and types of treatment used in the experiment.

	Spleen				Duodenum			
Treatment	Control	ZEA	*E coli*	ZEA + *E coli*	Control	ZEA	*E coli*	ZEA + *E coli*
Number of samples	4	3	4	4	3	4	4	4

The Agilent chips used for hybridization contained a custom panel of 60mer oligos specific for a large number of porcine transcripts (AMADID 056850 –Genotypic India) represented by 59,835 features. After scanning the microarray chips with the Agilent SureScan Microarray Scanner G2600D, the image processing was conducted using the Feature Extraction software, version 11.0.1.1, with grid 056850_D_F_20130729. The raw data that was obtained for each array was pre-processed, and submitted to ArrayExpress (Accesion Number: A-MTAB-556). The differential analysis was completed using the GeneSpring GX version 13.0 software developed by Agilent Technologies. Thus, control probes were eliminated, data were normalized using the quantile normalization method, and then various test were applied for each comparison. We calculated the fold change with a cut-off of 2.0, then we filtered on volcano plot, and applied a moderated *t-*test with False Discovery Rate (FDR) correction which limits the type I errors, in other words reduces the false positives to 5%. Consequently, we performed four comparisons for each of the two tested tissues (spleen and duodenum), according to the treatment that was previously administered: *E coli* vs control, ZEA vs control, and ZEA + *E coli* vs control, and ANOVA analysis, and for each we obtained a list of transcripts which presented a statistically significant differential expression. Clusters were formed, and the results were ready to undergo other types of functional tests, like pathway analysis (manuscript in preparation).

The pipeline was based on the LiftOver tool, which is capable of converting genome coordinates between different species. This tool is part of the Galaxy web based platform, which offers the infrastructure necessary for complete analyses for high throughput data experiments, data interrogation and integration via an accessible graphical user interface [9]. The input necessary for this program is a.*bed* file with the genome coordinates of the *Sus scrofa* probes. Therefore, the first step of this procedure was to subject the.*fasta* file that contained the sequences of the pig probes present on the microarray chip, to a series of conversions in order to obtain the file that was needed for the extrapolation. The.*fasta* file was converted to a.*fastq* file by using the *perl* script “*fasta_to_fastq*.*pl*” which was applied in an Ubuntu environment (Ubuntu 10.04.2), at command line. The.*fastq* file that was obtained contained a so-called “generic encoding”, unsuitable for the alignment tool, so it was necessary to transform it into a Sanger encoded format. The tool that was used was FastaGroomer 1.0.4, found under the “NGS: QC and manipulation” tab in the Galaxy suite [9], and the file that was generated had the following characteristic: Sanger scaled quality values with ASCII offset 33. It was now suitable for alignment, which was performed also with the help of a Galaxy tool. The sequences that needed to be matched to the porcine genome had a length of approximately 60 nucleotides each, so we used an alignment instrument that is specific for short reads, Bowtie 2, which also belongs to the Galaxy suite. The database that was used for the alignment of the probe sequences was susScr1, and the procedure generated a.*bam* file, and its corresponding.*bai*, containing the aligned probes, mapped to the *Sus scrofa* genome, and several other information among which the coordinates of the probes.

In order to obtain the human orthologues of the statistically significant differentially expressed porcine genes observed as a result of the microarray experiment, the genome coordinates of the transcripts was converted between the two species using the LiftOver 1.0.3 tool from Galaxy [9]. The required input file for this operation was a.*bed* format file, which was obtained in command line in Ubuntu, using *bamToBed*, a BEDTools suite sub-command, and the previously obtained.*bam* file as input [10]. The.*bed* file contains the coordinates of the *Sus scrofa* probe sequences, and it was used as input in the LiftOver 1.0.3 tool (Galaxy suite). The result of the conversion was also a.*bed* file which contains the genome coordinates of the human equivalent genes, aligned and mapped to *hg19*, as well as an additional file which contained the unmapped probe sequences, whose human counterparts could not be found. The file that contained the mapped genome coordinates was “groomed” in Excel (Microsoft Office 2013) in order to make it suitable for being used as input in the “Annotate Genomic Regions” freeware, variant May 2014, which was installed locally from the bioserver.iit.ieo.eu/AnnotateGenomicRegions/ website [11]. This tool, developed by computational researchers at the European School of Molecular Medicine, Italy, uses alignment to the human genome to add gene names to the corresponding coordinates, or, in our case, to add the equivalent human orthologue to the original porcine transcript probes. The version of the genome that was selected for the annotation of genomic region was *hg19*, which was compatible with all the tools used for the extrapolation, and the reference database was *RefGene*.

## Results and Discussions

**Fig 1:**
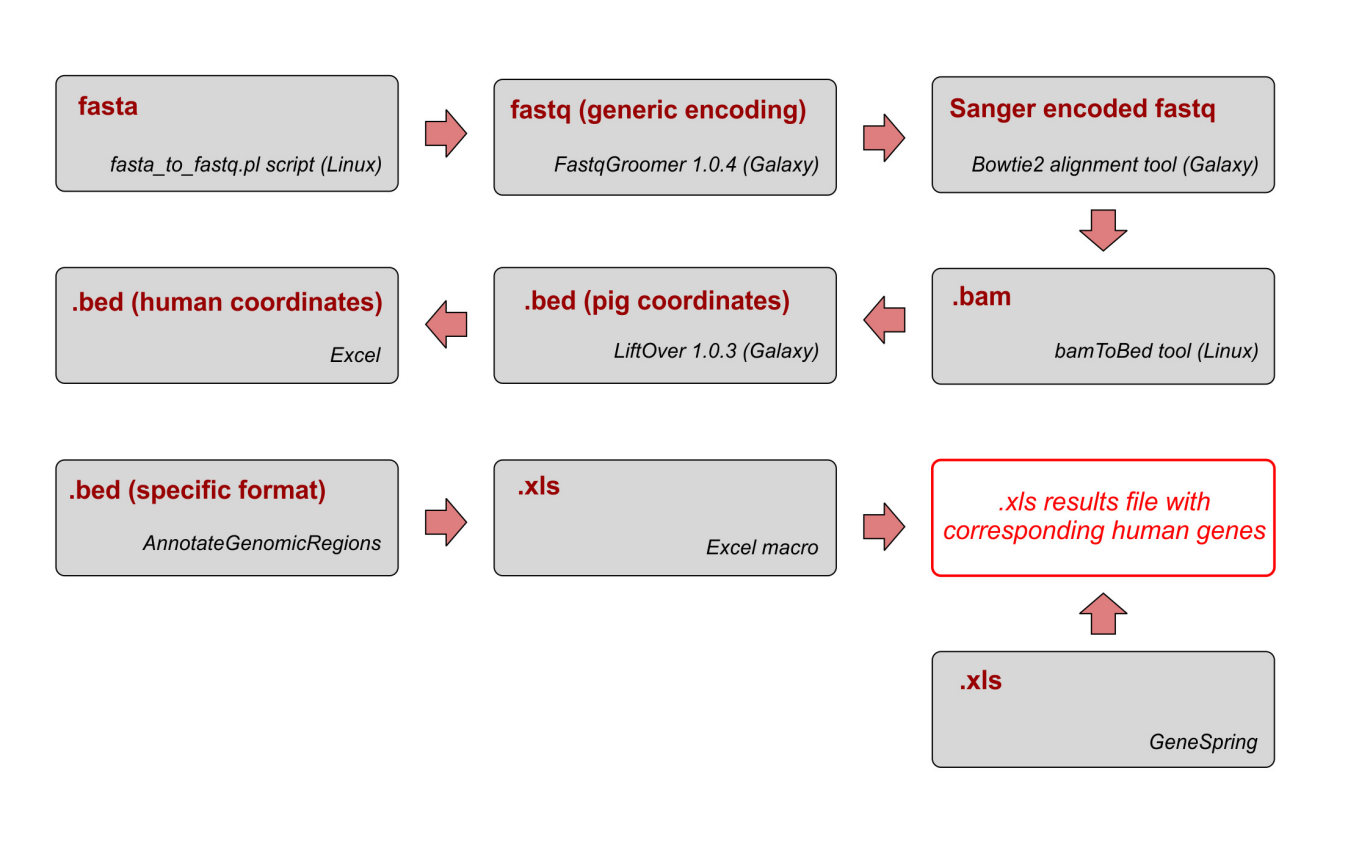
Pipeline used for the interspecies gene name extrapolation.

As a result of this procedure, which is depicted in Fig 1 and presented in more detail in S1 File. Extrapolation pipeline–step by step, we obtained a file which contained a list of the original probe IDs in one column and the corresponding human gene name in another. With the help of a “match and complete” macro, we further annotated each of the differentially expressed original porcine transcripts which were generated by GeneSpring analysis with their human equivalent gene name.

The pipeline that we developed proved to be a straight-forward and efficient tool for extrapolating gene names between species–in this case between *Sus scrofa* and *Homo sapiens*–when working with large custom panels which do not contain complete annotations or use less common nomenclatures. From the initial number of 59,835 porcine probes present on the microarray chip, the LiftOver tool aligned and mapped 40,860 regions to the human genome, while the other 18,976 could not be mapped, probably due to lack of homology. From the mapped regions, 110 were identified as belonging to the mitochondrial chromosome, and were not taken into consideration. The rest of the transcript coordinates (40,750) were used as input query into the annotation tool, and 36,937 were annotated with the equivalent human RefGene symbol. A further analysis revealed that these annotations referred to 13,182 individual gene names. Thus, from the original 59,835 *Sus scrofa* probes, 36,937 were translated into human orthologues, which represents a yield of approximately 62%.

To validate the efficiency and the practical utility of our method, we performed a comparative search, on a smaller scale, using the BioMart tool from Ensembl. We took the statistically significant differentially expressed transcripts for each of the analysis groups for the two tissue types (duodenum and spleen–E coli vs control, ZEA vs control, ZEA + E coli vs control and ANOVA) and ran them through BioMart. The efficiency of probe name detection for the original porcine transcript IDs ranged between 0.67% and 5.67%, with an average of 3.64%. As a comparison, counting the extrapolated human genes in the results of the same analysis groups returned values between 52.48% and 63.47%, with an average of 58.34%. The complete query result values are presented in Table 2.

**Table 2:** The performance of the novel gene name extrapolation method, compared to web-based tools.

Tissue	Differential expression analysis	*Sus scrofa*	*Homo sapiens*			
		Probe IDs	Biomart		The extrapolation pipeline	
			Count	%	Count	%
Duodenum	E coli_vs_ctrl	2875	128	4.45	1679	58.40
	Zea_vs_ctrl	4023	148	3.68	2457	61.07
	Zea_E coli_vs_ctrl	316	13	4.11	172	54.43
	Anova	296	15	5.07	171	57.77
Spleen	E coli_vs_ctrl	1649	19	1.15	960	58.22
	Zea_vs_ctrl	141	8	5.67	74	52.48
	Zea_E coli_vs_ctrl	23	1	4.35	14	60.87
	Anova	1489	10	0.67	945	63.47

## Conclusions

Consequently, while online data analysis portals are of indisputable importance and value by facilitating biological data management and assessment, our particular situation needed a more complex, trans-species approach, which proved to generate useful results. Thus, we were able to identify the human orthologues which are differentially expressed in a statistically significant manner, increasing our understanding of how co-contamination might affect both pigs and humans, two species which share many anatomical, physiological, dietary and environmental similarities. In the “omics” era, when similar situations can be often encountered by researcher using various animal models, our new animal-to-human gene name extrapolation method offers an innovative solution for efficient data analysis and interpretation.

## Supporting Information

S1 File Extrapolation pipeline–step by step. (PDF)

## References

[1] Cekanova M, Rathore K. Animal models and therapeutic molecular targets of cancer: utility and limitations. Drug design, development and therapy. 2014;8:1911–21. 10.2147/DDDT.S49584 25342884 PMC4206199

[2] Tenaillon O, Skurnik D, Picard B, Denamur E. The population genetics of commensal Escherichia coli. Nature reviews Microbiology. 2010;8(3):207–17. 10.1038/nrmicro2298 . 20157339

[3] Sekirov I, Russell SL, Antunes LC, Finlay BB. Gut microbiota in health and disease. Physiological reviews. 2010;90(3):859–904. 10.1152/physrev.00045.2009 . 20664075

[4] Huffnagle G, Noverr MC. GI microbiota and regulation of the immune system. Preface. Advances in experimental medicine and biology. 2008;635:v–vi. . 18841698

[5] Kollarczik B, Gareis M, Hanelt M. In vitro transformation of the Fusarium mycotoxins deoxynivalenol and zearalenone by the normal gut microflora of pigs. Natural toxins. 1994;2(3):105–10. . 8087428 10.1002/nt.2620020303

[6] Piotrowska M, Slizewska K, Nowak A, Zielonka L, Zakowska Z, Gajecka M, et al The effect of experimental fusarium mycotoxicosis on microbiota diversity in porcine ascending colon contents. Toxins. 2014;6(7):2064–81. 10.3390/toxins6072064 25025709 PMC4113742

[7] Kinsella RJ, Kahari A, Haider S, Zamora J, Proctor G, Spudich G, et al Ensembl BioMarts: a hub for data retrieval across taxonomic space. Database: the journal of biological databases and curation. 2011;2011:bar030 10.1093/database/bar030 21785142 PMC3170168

[8] Cunningham F, Amode MR, Barrell D, Beal K, Billis K, Brent S, et al Ensembl 2015. Nucleic acids research. 2015;43(Database issue):D662–9. 10.1093/nar/gku1010 . 25352552 PMC4383879

[9] Blankenberg D, Von Kuster G, Coraor N, Ananda G, Lazarus R, Mangan M, et al Galaxy: a web-based genome analysis tool for experimentalists. Current protocols in molecular biology / edited by Ausubel Frederick M [et al]. 2010;Chapter 19:Unit 19 0 1–21. 10.1002/0471142727.mb1910s89 20069535 PMC4264107

[10] Quinlan AR, Hall IM. BEDTools: a flexible suite of utilities for comparing genomic features. Bioinformatics. 2010;26(6):841–2. 10.1093/bioinformatics/btq033 20110278 PMC2832824

[11] Zammataro L, DeMolfetta R, Bucci G, Ceol A, Muller H. AnnotateGenomicRegions: a web application. BMC bioinformatics. 2014;15 Suppl 1:S8 10.1186/1471-2105-15-S1-S8 24564446 PMC4015944

