# Supplementary material for: Interspecies Gene Name Extrapolation—A New Approach
Source: PLoS One. 2015 Sep 25;10(9):e0138751. doi: 10.1371/journal.pone.0138751 (PMC4583432; doi:10.1371/journal.pone.0138751)
Supplement: S1 File — (PDF) [file pone.0138751.s001.pdf]

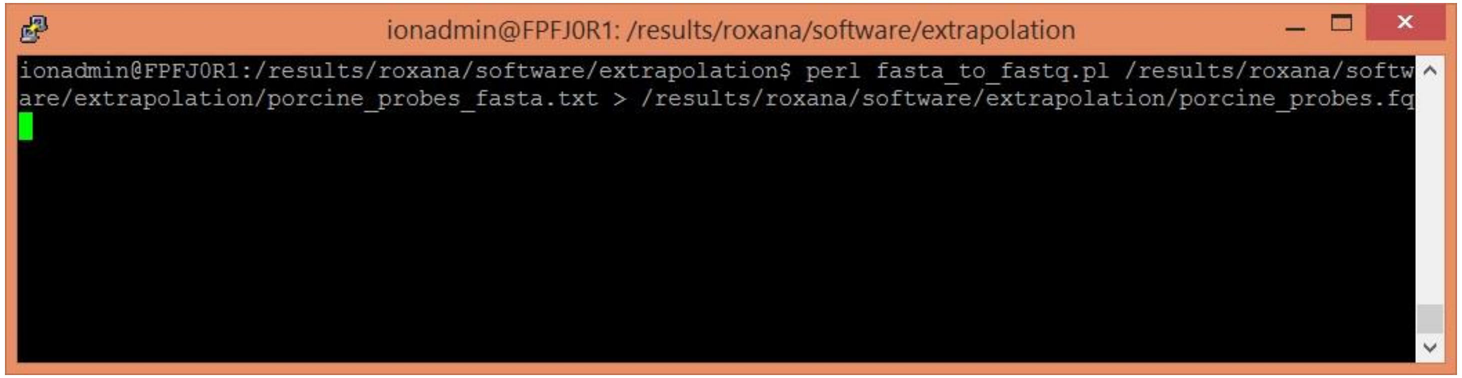

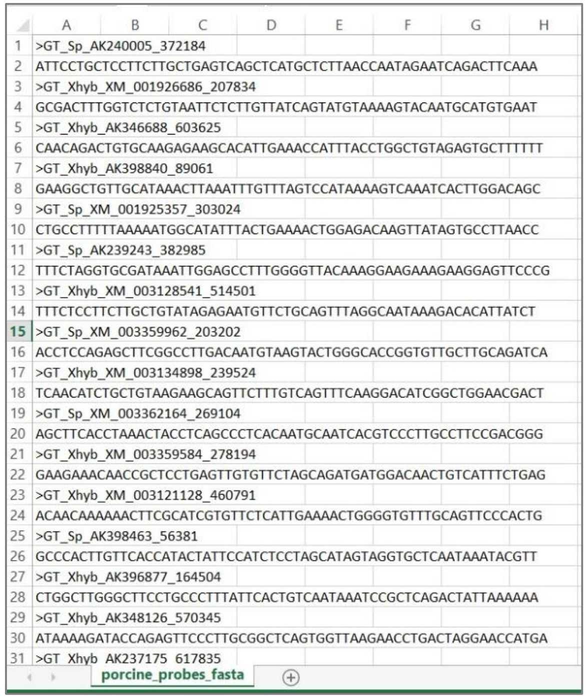

	A
1	>GT_Sp_AK240005_372184
2	ATTCCTGCTCCTTCTTGCTGAGTCAGCTCATGCTCTTAACCAATAGAATCAGACTTCAAA
3	>GT_Xhyb_XM_001926686_207834
4	GCGACTTTGGTCTCTGTAATTCTCTTGTTATCAGTATGTAAAAGTACAATGCATGTGAAT
5	>GT_Xhyb_AK346688_603625
6	CAACAGACTGTGCAAGAGAAGCACATTGAAACCATTTACCTGGCTGTAGAGTGCTTTTTT
7	>GT_Xhyb_AK398840_89061
8	GAAGGCTGTTGCATAAACTTAAATTTGTTTAGTCCATAAAAGTCAAATCACTTGGACAGC
9	>GT_Sp_XM_001925357_303024
10	CTGCCTTTTTAAAAATGGCATATTTACTGAAAACTGGAGACAAGTTATAGTGCCTTAACC
11	>GT_Sp_AK239243_382985
12	TTTCTAGGTGCGATAAATTGGAGCCTTTGGGGTTACAAAGGAAGAAAGAAGGAGTTCCCG
13	>GT_Xhyb_XM_003128541_514501
14	TTTCTCCTTCTTGCTGTATAGAGAATGTTCTGCAGTTTAGGCAATAAAGACACATTATCT
15	>GT_Sp_XM_003359962_203202
16	ACCTCCAGAGCTTCGGCCTTGACAATGTAAGTACTGGGCACCGGTGTTGCTTGCAGATCA
17	>GT_Xhyb_XM_003134898_239524
18	TCAACATCTGCTGTAAGAAGCAGTTCTTTGTCAGTTTCAAGGACATCGGCTGGAACGACT
19	>GT_Sp_XM_003362164_269104
20	AGCTTCACCTAAACTACCTCAGCCCTCACAATGCAATCACGTCCCTTGCCTTCCGACGGG
21	>GT_Xhyb_XM_003359584_278194
22	GAAGAAACAACCGCTCCTGAGTTGTGTTCTAGCAGATGATGGACAACTGTCATTTCTGAG
23	>GT_Xhyb_XM_003121128_460791
24	ACAACAAAAAACTTCGCATCGTGTTCTCATTGAAAACTGGGGTGTTTGCAGTTCCCACTG
25	>GT_Sp_AK398463_56381
26	GCCCACTTGTTCACCATACTATTCCATCTCCTAGCATAGTAGGTGCTCAATAAATACGTT
27	>GT_Xhyb_AK396877_164504
28	CTGGCTTGGGCTTCCTGCCCTTTATTCACTGTCAATAAATCCGCTCAGACTATTAAAAAA
29	>GT_Xhyb_AK348126_570345
30	ATAAAAGATACCAGAGTTCCCTTGCGGCTCAGTGGTTAAGAACCTGACTAGGAACCATGA
31	>GT_Xhyb_AK237175_617835

porcine_probes_fasta

fasta

fastq

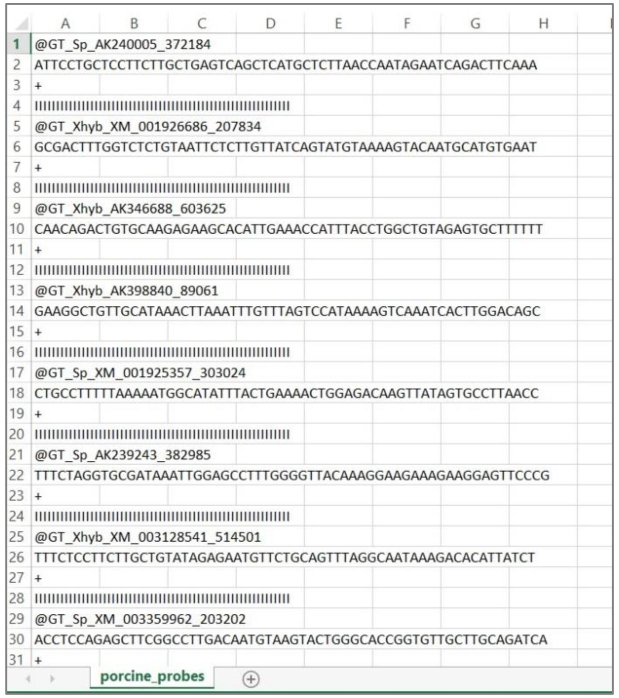

	A
1	@GT_Sp_AK240005_372184
2	ATTCCTGCTCCTTCTTGCTGAGTCAGCTCATGCTCTTAACCAATAGAATCAGACTTCAAA
3	+
4	IIIIIIIIIIIIIIIIIIIIIIIIIIIIIIIIIIIIIIIIIIIIIIIIIII
5	@GT_Xhyb_XM_001926686_207834
6	GCGACTTTGGTCTCTGTAATTCTCTTGTTATCAGTATGTAAAAGTACAATGCATGTGAAT
7	+
8	IIIIIIIIIIIIIIIIIIIIIIIIIIIIIIIIIIIIIIIIIIIIIIIIIII
9	@GT_Xhyb_AK346688_603625
10	CAACAGACTGTGCAAGAGAAGCACATTGAAACCATTTACCTGGCTGTAGAGTGCTTTTTT
11	+
12	IIIIIIIIIIIIIIIIIIIIIIIIIIIIIIIIIIIIIIIIIIIIIIIIIII
13	@GT_Xhyb_AK398840_89061
14	GAAGGCTGTTGCATAAACTTAAATTTGTTTAGTCCATAAAAGTCAAATCACTTGGACAGC
15	+
16	IIIIIIIIIIIIIIIIIIIIIIIIIIIIIIIIIIIIIIIIIIIIIIIIIII
17	@GT_Sp_XM_001925357_303024
18	CTGCCTTTTTAAAAATGGCATATTTACTGAAAACTGGAGACAAGTTATAGTGCCTTAACC
19	+
20	IIIIIIIIIIIIIIIIIIIIIIIIIIIIIIIIIIIIIIIIIIIIIIIIIII
21	@GT_Sp_AK239243_382985
22	TTTCTAGGTGCGATAAATTGGAGCCTTTGGGGTTACAAAGGAAGAAAGAAGGAGTTCCCG
23	+
24	IIIIIIIIIIIIIIIIIIIIIIIIIIIIIIIIIIIIIIIIIIIIIIIIIII
25	@GT_Xhyb_XM_003128541_514501
26	TTTCTCCTTCTTGCTGTATAGAGAATGTTCTGCAGTTTAGGCAATAAAGACACATTATCT
27	+
28	IIIIIIIIIIIIIIIIIIIIIIIIIIIIIIIIIIIIIIIIIIIIIIIIIII
29	@GT_Sp_XM_003359962_203202
30	ACCTCCAGAGCTTCGGCCTTGACAATGTAAGTACTGGGCACCGGTGTTGCTTGCAGATCA
31	+

porcine_probes

fasta

- *fasta_to_fastq.pl script (Linux)*

FASTQ Groomer - Galaxy

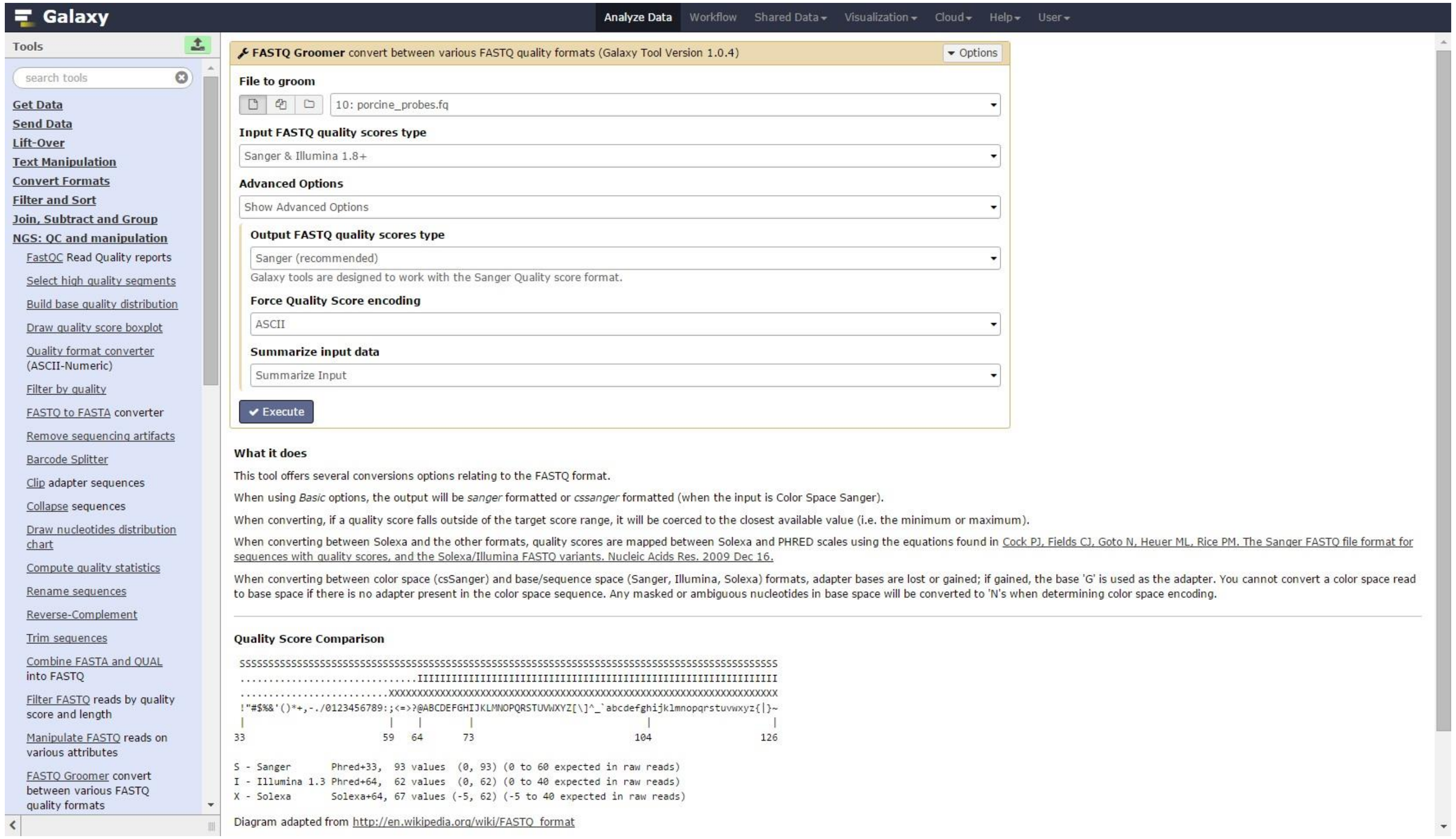

fastq (generic encoding)

- *FastqGroomer 1.0.4 (Galaxy)*

Bowtie2 Alignment Tool - Galaxy

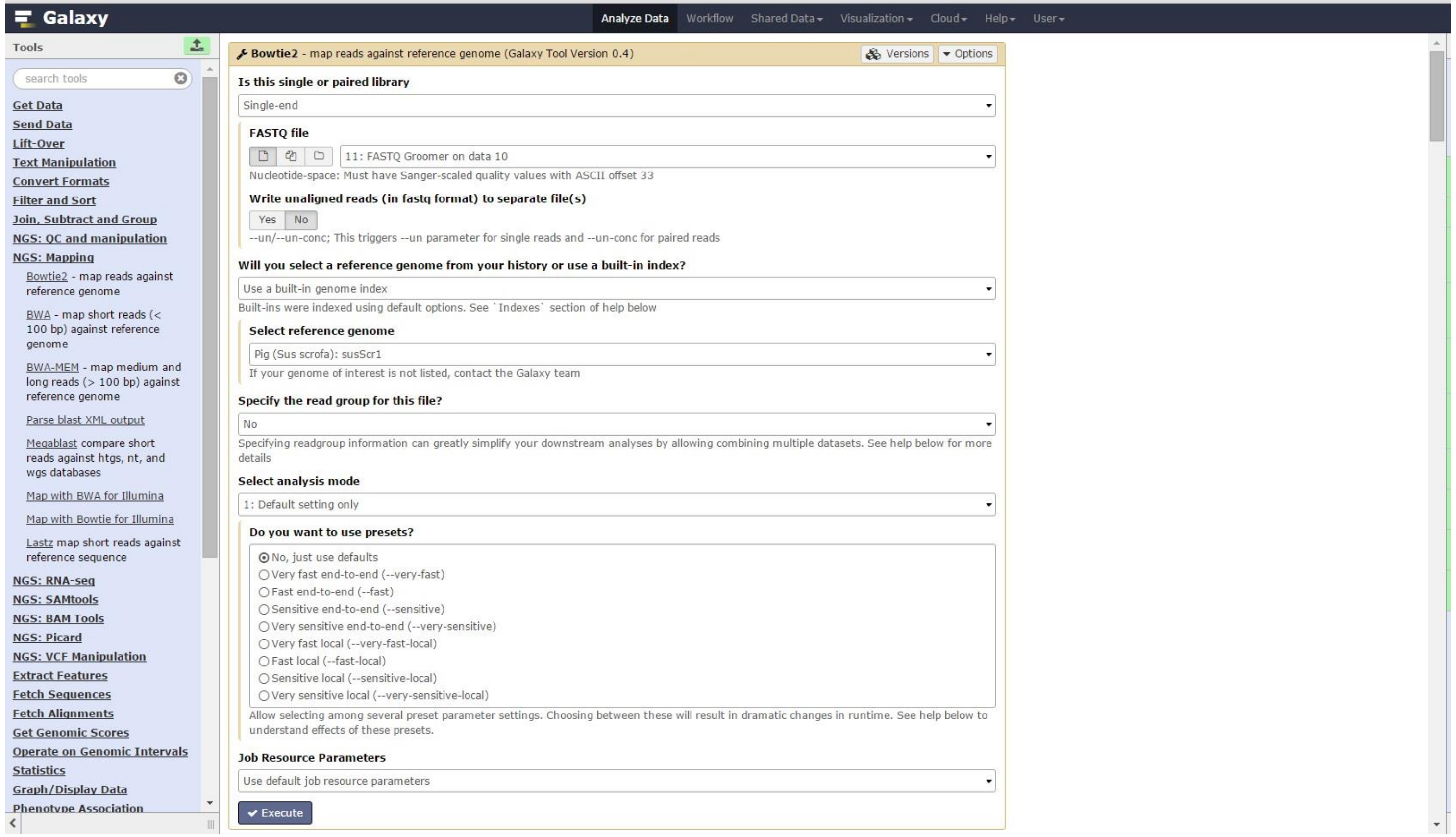

Sanger encoded fastq

- *Bowtie2 alignment tool (Galaxy)*

```
ionadmin@FPFJ0R1: /results/roxana/software/extrapolation
ionadmin@FPFJ0R1:/results/roxana/software/samtools-1.1$ bamToBed -i /results/roxana/software/extrapolation/aligned_po
rcine_reads.bam > /results/roxana/software/extrapolation/aligned_porcine_reads.bed
ionadmin@FPFJ0R1:/results/roxana/software/samtools-1.1$ cd /results/roxana/software/extrapolation/
ionadmin@FPFJ0R1:/results/roxana/software/extrapolation$ ls -l
total 18288
-rw-r--r-- 1 ionadmin ionadmin 2137149 Aug  4 21:17 aligned_porcine_reads.bam
-rw-r--r-- 1 ionadmin ionadmin 2402284 Aug  4 21:22 aligned_porcine_reads.bed
-rw-r--r-- 1 ionadmin ionadmin 8955318 Aug  4 20:12 porcine_probes.fq
-rw-r--r-- 1 ionadmin ionadmin 5185713 Aug  4 19:56 porcine_probes_fasta.txt
ionadmin@FPFJ0R1:/results/roxana/software/extrapolation$
```

	A	B	C	D	E	F	G
1	chr1	366332	366392	GT_Sp_AK395166_101321	1	+	
2	chr1	388606	388666	GT_Xhyb_XM_003121080_268774	1	-	
3	chr1	538513	538573	GT_Sp_XM_003121082_236224	42	-	
4	chr1	580869	580929	GT_Sp_XM_003353155_268764	1	+	
5	chr1	630318	630378	GT_Xhyb_AK346362_532521	1	-	
6	chr1	630321	630381	GT_Xhyb_AK347679_582265	1	-	
7	chr1	630321	630381	GT_Xhyb_AK396242_64421	1	-	
8	chr1	630964	631024	GT_Xhyb_AK392154_101461	1	+	
9	chr1	630964	631024	GT_Xhyb_AK236266_333174	1	+	
10	chr1	631133	631193	GT_Xhyb_XM_003121076_236244	1	-	
11	chr1	631133	631193	GT_Xhyb_XM_003121078_236234	1	-	
12	chr1	631446	631506	GT_Xhyb_XM_003480211_268784	1	+	
13	chr1	1039503	1039563	GT_Xhyb_XM_003121075_236214	42	-	
14	chr1	1039503	1039563	GT_Xhyb_AK352101_618065	42	-	
15	chr1	1331076	1331136	GT_Sp_XM_003121083_268754	1	-	
16	chr1	1337873	1337933	GT_Sp_XM_003480213_187404	1	-	
17	chr1	1405876	1405936	GT_Xhyb_XM_003480215_187394	1	-	
18	chr1	1505418	1505478	GT_Sp_XM_003480214_203164	32	-	
19	chr1	1757180	1757240	GT_Sp_XM_003121086_319744	1	-	
20	chr1	2065305	2065365	GT_Xhyb_XM_003121091_482861	42	-	
21	chr1	2108353	2108413	GT_Sp_XM_003121094_535211	42	+	
22	chr1	2129786	2129842	GT_Sp_XM_003121093_299594	0	+	
23	chr1	2190399	2190459	GT_Sp_XM_003121087_319734	42	+	
24	chr1	2456147	2456207	GT_Xhyb_XM_003121095_299584	42	-	
25	chr1	2515405	2515465	GT_Sp_XM_001928144_268753	1	-	
26	chr1	2631627	2631687	GT_Xhyb_AK392532_98771	1	-	
27	chr1	2631627	2631687	GT_Xhyb_NM_001244534_11141	1	-	
28	chr1	2639067	2639127	GT_Sp_AK392262_159384	1	-	
29	chr1	2648119	2648179	GT_Sp_XM_003353160_236204	42	-	
30	chr1	2657770	2657830	GT_Xhyb_AK400085_58311	1	-	
31	chr1	2657770	2657830	GT_Xhyb_AK234386_410184	1	-	

aligned_porcine_reads

bed file

.bam

- *bamToBed tool (Linux)*

LiftOver Tool - Galaxy

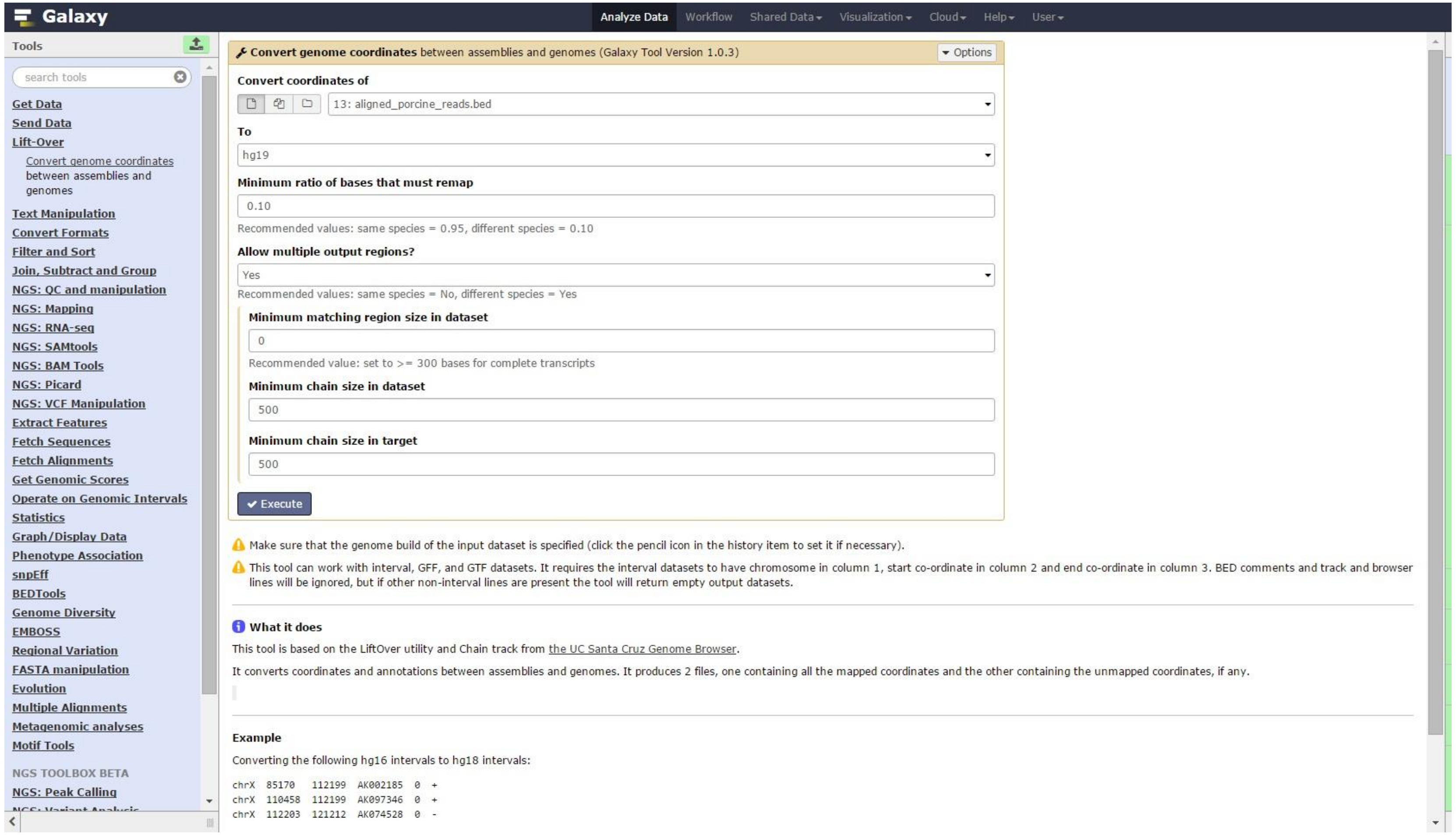

.bed (pig coordinates)

- *LiftOver 1.0.3 (Galaxy)*

	A	B	C	D	E	F	G
1	chr6	170128123	170128184	GT_Sp_AK395166_101321	1	-	
2	chr6	170105107	170105169	GT_Xhyb_XM_003121080_268774	1	+	
3	chr6	170181645	170181706	GT_Sp_XM_003121082_236224	1	+	
4	chr6	170036527	170036587	GT_Sp_XM_003353155_268764	1	-	
5	chr6	170104032	170104083	GT_Xhyb_AK346362_532521	1	-	
6	chr6	170104033	170104086	GT_Xhyb_AK347679_582265	1	-	
7	chr6	170104033	170104086	GT_Xhyb_AK396242_64421	1	-	
8	chr6	170104639	170104689	GT_Xhyb_AK392154_101461	1	+	
9	chr6	170104639	170104689	GT_Xhyb_AK236266_333174	1	+	
10	chr6	170104800	170104864	GT_Xhyb_XM_003121076_236244	1	-	
11	chr6	170104800	170104864	GT_Xhyb_XM_003121078_236234	1	-	
12	chr6	170105121	170105179	GT_Xhyb_XM_003480211_268784	1	+	
13	chr6	169615905	169615963	GT_Xhyb_XM_003121075_236214	1	-	
14	chr6	169615905	169615963	GT_Xhyb_AK352101_618065	1	-	
15	chr6	169067128	169067193	GT_Sp_XM_003480213_187404	1	+	
16	chr6	168949809	168949869	GT_Xhyb_XM_003480215_187394	1	+	
17	chr6	168708111	168708171	GT_Sp_XM_003121086_319744	1	-	
18	chr6	168457776	168457859	GT_Xhyb_XM_003121091_482861	1	-	
19	chr6	168372462	168372522	GT_Sp_XM_003121094_535211	1	+	
20	chr6	168405230	168405315	GT_Sp_XM_003121093_299594	1	+	
21	chr6	168206146	168206210	GT_Sp_XM_003121087_319734	1	-	
22	chr6	167717486	167717546	GT_Xhyb_XM_003121095_299584	1	+	
23	chr6	166579249	166579309	GT_Sp_XM_001928144_268753	1	-	
24	chr6	166733357	166733413	GT_Xhyb_AK392532_98771	1	-	
25	chr6	166733357	166733413	GT_Xhyb_NM_001244534_11141	1	-	
26	chr6	166719827	166719885	GT_Sp_XM_003353160_236204	1	-	
27	chr6	166733357	166733413	GT_Xhyb_AK400085_58311	1	-	
28	chr6	166733357	166733413	GT_Xhyb_AK234386_410184	1	-	
29	chr6	166778442	166778508	GT_Xhyb_AK391807_103151	1	-	
30	chr6	166778442	166778508	GT_Xhyb_AK399856_62441	1	-	
31	chr6	166778471	166778538	GT_Xhyb_AK396434_119645	1	-	

MAPPED_COORDINATES

Raw mapped coordinates

.bed (human coordinates)

- *Excel*

.bed (specific format)

- *AnnotateGenomicRegions*

Groomed mapped coordinates

	A	B	C	D	E
1	chr6:170104800-170104864				
2	chr6:170104639-170104689				
3	chr6:170104033-170104086				
4	chr6:170036527-170036587				
5	chr6:170181645-170181706				
6	chr6:170104032-170104083				
7	chr6:170104033-170104086				
8	chr6:170104639-170104689				
9	chr6:170104800-170104864				
10	chr6:170105107-170105169				
11	chr6:170105121-170105179				
12	chr6:170128123-170128184				
13	chr6:169615905-169615963				
14	chr6:169615905-169615963				
15	chr6:169067128-169067193				
16	chr6:168949809-168949869				
17	chr6:168708111-168708171				
18	chr6:168457776-168457859				
19	chr6:168372462-168372522				
20	chr6:168405230-168405315				
21	chr6:168206146-168206210				
22	chr6:167717486-167717546				
23	chr6:166733357-166733413				
24	chr6:166719827-166719885				
25	chr6:166733357-166733413				
26	chr6:166733357-166733413				
27	chr6:166733357-166733413				
28	chr6:166778442-166778508				
29	chr6:166778442-166778508				
30	chr6:166778471-166778538				
31	chr6:166827278-166827338				

mapped_gene_location

“Annotate Genomic Regions” Tool

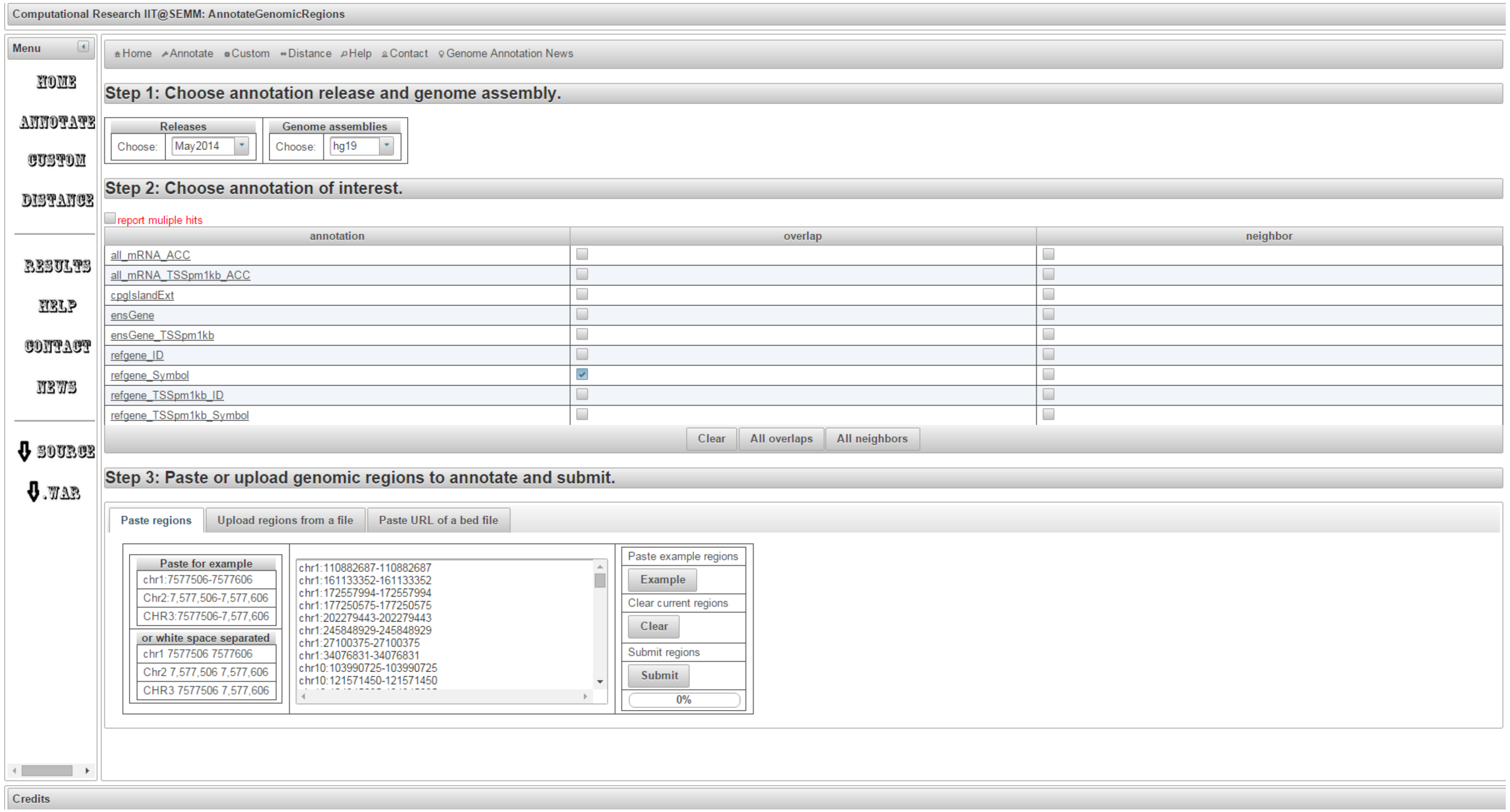

	A	B	C	D	E	F	G
1	ProbeID (pig)	Symbol (HGNC)	CHR	Start	Stop	Strand	
2	GT_Sp_AK235946_388634	PALMD	chr1	100133412	100133829	+	
3	GT_Xhyb_DQ322458_467001	PALMD	chr1	100159842	100159900	+	
4	GT_Xhyb_AK343908_591815	PALMD	chr1	100159842	100159900	+	
5	GT_Xhyb_NM_001038645_1611	PALMD	chr1	100159842	100159900	+	
6	GT_Sp_EU647218_575755	MIR548AA1	chr1	100174576	100174636	-	
7	GT_Xhyb_AK345185_502771	AGL	chr1	100330110	100330170	+	
8	GT_Sp_XM_003481507_198194	AGL	chr1	100389513	100389574	+	
9	GT_Xhyb_DQ883629_424221	SLC35A3	chr1	100487972	100488032	+	
10	GT_Sp_AK343998_613945	HIAT1	chr1	100525837	100525900	+	
11	GT_Sp_AK390584_132174	TRMT13	chr1	100599727	100599790	+	
12	GT_Sp_AK344506_620706	TRMT13	chr1	100608410	100608470	+	
13	GT_Xhyb_XM_003361695_270814	TRMT13	chr1	100613842	100613903	-	
14	GT_Xhyb_AY610221_432751	TRMT13	chr1	100613842	100613903	-	
15	GT_Xhyb_XM_003481505_198204	DBT	chr1	100661520	100661534	-	
16	GT_Xhyb_XM_001924840_227034	DBT	chr1	100661520	100661534	-	
17	GT_Xhyb_AK393486_86921	DBT	chr1	100713227	100713289	-	
18	GT_Xhyb_AK346352_460411	DBT	chr1	100713228	100713290	-	
19	GT_Xhyb_AK235362_347424	RTCA	chr1	100757036	100757096	+	
20	GT_Xhyb_AK394808_169034	RTCA	chr1	100757227	100757285	+	
21	GT_Xhyb_NM_001145222_1421	RBP7	chr1	10075924	10075984	+	
22	GT_Xhyb_EF208119_559775	RBP7	chr1	10075924	10075984	+	
23	GT_Xhyb_AY609848_434611	RBP7	chr1	10075932	10075992	+	
24	GT_Xhyb_XM_003125892_259554	CDC14A	chr1	100819348	100819408	+	
25	GT_Sp_AK350122_626374	CDC14A	chr1	100849111	100849171	+	
26	GT_Xhyb_AK350812_482921	CDC14A	chr1	100905503	100905563	+	
27	GT_Sp_XM_003125888_259564	CDC14A	chr1	100963673	100963733	+	
28	GT_Sp_XM_003355282_227054	GPR88	chr1	101005985	101006048	+	
29	GT_Xhyb_L43124_511091	VCAM1	chr1	101204146	101204202	+	
30	GT_Xhyb_AK235268_347894	VCAM1	chr1	101204490	101204550	+	
31	GT_Xhyb_AK391233_170134	VCAM1	chr1	101204490	101204550	+	

annotations_Pig_to_hg19

	A	B	C	D	E	F	G
1		# Notes : Created from Advanced Analysis operation: significance Analysis.					
2		#Entitylist : [Ecd] vs [Cd] Fold change >= 2.0					
3		#Interpretation : TRAT ecd vs cd					
4		#Experiment: duodenum R1 Q					
5		#corrected p-value cut-off:0.05					
6		#Fold change cut-off:2.0					
7		#Selected Test : Moderated T-Test					
8		#p-value computation: Asymptotic					
9		#Multiple Testing Correction: Benjamini-Hochberg					
10		# Technology : Agilent.SingleColor.56850					
11		# Owner : gxuser					
12							
13		**ProbeName**	**p (Corr)**	**p**	**FC (abs)**	**Regulation**	**[Cd](normalized)**
14	1	GT_Xhyb_AY610329_432211	0.01002455	0.00426551	2.513669	up	10.900504
15	2	GT_Xhyb_AF455037_547547	0.01011821	0.00431769	2.04365	up	5.9715257
16	3	GT_Xhyb_AK240565_357604	0.00189811	1.52E-04	2.350802	down	8.461801
17	4	GT_Xhyb_XM003133379_4583[illegible]	0.00187544	1.41E-04	2.334001	down	12.91364
18	5	GT_Xhyb_XM003361558_2713[illegible]	0.00176953	1.06E-04	2.732703	down	6.606388
19	6	GT_Xhyb_XM003354024_23187	0.00171076	6.48E-05	2.575319	down	8.723351
20	7	GT_Xhyb_KC333253_35931	0.01050231	0.00457891	2.557626	up	4.515629
21	8	GT_Xhyb_AK349076_549147	0.04166641	0.02794017	2.016263	up	11.176852
22	9	GT_Xhyb_AK399314_134004	0.001998	1.94E-04	3.250202	up	14.243794
23	10	GT_Sp_XM003354059_232074	0.00749414	0.00277764	4.378521	up	2.4877229
24	11	GT_Xhyb_XM003130668_2710[illegible]	0.00585521	0.0017998	2.075772	up	8.004399
25	12	GT_Xhyb_AK397931_123174	0.01322491	0.00623354	2.017953	up	11.374532
26	13	GT_Xhyb_AY609994_433881	0.00491607	0.00131554	2.043104	up	13.227538
27	14	GT_Sp_AK398262_118545	0.00686761	0.00240643	2.163662	down	11.644561
28	15	GT_Xhyb_XM003130846_2837[illegible]	0.0050415	0.00137411	3.704003	up	5.8021913
29	16	GT_Sp_XM001928648_512001	0.00657339	0.00222312	2.168653	down	11.209969
30	17	GT_Xhyb_XM003123137_5345[illegible]	0.00186374	1.38E-04	2.492698	down	10.483933
31	18	GT_Xhyb_AK231130_356824	0.00305916	5.17E-04	2.129779	down	13.491168
32	19	GT_Xhyb_AK238486_343094	0.04151525	0.02778819	3.680049	down	5.073385
33	20	GT_Xhyb_AK350622_571975	0.01431285	0.00692084	2.021152	up	13.085031
34	21	GT_Xhyb_XM003124963_2939[illegible]	0.00341759	6.45E-04	2.380063	up	8.448777

ecd_vs_control | cluster

EXCEL macro

	A	B	C	D	E	F	G
1	# Notes : Created from Advanced Analysis operation: significance Analysis.						
2	#Entitylist : [Ecd] vs [Cd] Fold change >= 2.0						
3	#Interpretation : TRAT ecd vs cd						
4	#Experiment: duodenum R1 Q						
5	#corrected p-value cut-off:0.05						
6	#Fold change cut-off:2.0						
7	#Selected Test : Moderated T-Test						
8	#p-value computation: Asymptotic						
9	#Multiple Testing Correction: Benjamini-Hochberg						
10	# Technology : Agilent.SingleColor.56850						
11							
12	**ProbeName**	**PredictedInHuman**	**p (Corr)**	**p**	**FC (abs)**	**Regulation**	**[Cd](normalized)[**
13	GT_Xhyb_XM001926938_2079[illegible]	**AAR2**	0.00431685	0.00102902	2.787506	down	7.221489
14	GT_Xhyb_XM003135473_3283[illegible]	**ABCD1**	0.01882937	0.01003682	2.221747	down	3.6280515
15	GT_Xhyb_AK396622_55571	**ABCG2**	0.04140772	0.02764553	2.767698	down	5.62764
16	GT_Sp_AK239782_555388	**ABHD2**	0.01635868	0.00824117	3.087869	down	5.0371103
17	GT_Xhyb_AK394436_120765	**ABLIM1**	0.02139165	0.0117834	2.645007	down	4.363725
18	GT_Xhyb_XM001928409_22241	**ABT1**	0.00585521	0.00179064	2.54671	down	5.0918922
19	GT_Xhyb_AB527055_619185	**ACSL1**	0.00230038	2.71E-04	2.514066	down	3.4212167
20	GT_Xhyb_XM003482282_17927	**ACTN1**	0.00619149	0.0019823	2.162534	up	4.8683634
21	GT_Xhyb_AK389443_94361	**ACTR1A**	0.00230455	2.74E-04	2.26156	down	8.483933
22	GT_Xhyb_HQ180176_494411	**ACVR1**	0.00171076	6.03E-05	2.048185	up	6.199241
23	GT_Sp_AK343296_590195	**ACVRL1**	0.02212823	0.01228627	2.207867	down	4.7475204
24	GT_Xhyb_AF069648_454381	**ADAM17**	0.00462705	0.00116776	2.047657	up	4.6502633
25	GT_Sp_XM003134106_208734	**ADAM19**	0.00646751	0.00215865	2.760812	up	5.0357337
26	GT_Sp_XM003132747_212784	**ADAMTS5**	0.04145658	0.02771859	2.876919	down	5.053725
27	GT_Sp_AK398031_122688	**ADAMTS9**	0.00824408	0.00323853	2.330393	down	4.3142276
28	GT_Sp_AK350858_531122	**ADAMTSL3**	0.03469672	0.02202282	2.032077	down	3.114149
29	GT_Sp_XM003125799_313214	**ADAMTSL4**	0.01477018	0.00721041	2.110519	down	4.8576965
30	GT_Sp_AK348045_617545	**ADCY4**	0.02866976	0.01707182	2.105845	down	6.626921
31	GT_Sp_XM003354632_262574	**ADCY9**	0.02427916	0.01378246	2.030168	up	4.0192156
32	GT_Xhyb_XM003128818_44747	**ADD1**	0.00302896	5.03E-04	2.343925	up	6.218474
33	GT_Sp_XM003122295_233424	**ADNP2**	0.04041052	0.02686152	2.518728	down	3.783395
34	GT_Sp_GACC01000199_42981	**ADO**	0.0032109	5.69E-04	2.008186	up	8.08294

ecd_vs_control | cluster

.xls results file with human orthologues
